# The Anti-Glioblastoma Effects of Novel Liposomal Formulations Loaded with Cannabidiol, Celecoxib, and 2,5-Dimethylcelecoxib

**DOI:** 10.3390/pharmaceutics17081031

**Published:** 2025-08-08

**Authors:** Anna Rybarczyk, Aleksandra Majchrzak-Celińska, Ludwika Piwowarczyk, Violetta Krajka-Kuźniak

**Affiliations:** 1 Chair and Department of Pharmaceutical Biochemistry, Poznan University of Medical Sciences, Rokietnicka 3, 60-806 Poznań, Poland; anna.rybarczyk@student.ump.edu.pl (A.R.); majchrzakcelinska@ump.edu.pl (A.M.-C.); 2 Doctoral School, Poznan University of Medical Sciences, Bukowska 70, 60-812 Poznań, Poland; 3 Chair and Department of Pharmaceutical Chemistry, Poznan University of Medical Sciences, Rokietnicka 3, 60-806 Poznań, Poland; lpiwowarczyk@ump.edu.pl

**Keywords:** Glioblastoma (GBM), cannabidiol (CBD), celecoxib (CELE), 2,5-dimethylcelecoxib (DMC), nanoformulation, liposomes, combinatorial treatment, Wnt/β-catenin pathway, NF-κB pathway, Nrf2 pathway

## Abstract

**Background/Objectives**: Glioblastoma multiforme (GBM) therapy efficacy remains limited due to the poor blood-brain barrier-penetrating power of drugs as well as dysregulated cellular signaling pathways of tumor cells leading to drug resistance. Novel drug delivery systems such as liposome-based nanoformulations improve the bioavailability and stability of water-insoluble drugs, while co-delivery of two anti-cancer compounds can further increase their anti-tumor effectiveness due to synergistic effects. Thus, the aim of this study was to obtain liposomal nanoformulations encapsulating cannabidiol (CBD), celecoxib (CELE), and 2,5-dimethylcelecoxib (DMC) and their combinations and to verify their anti-GBM properties. **Methods**: Five liposomal nanoformulations were obtained using a modified thin-film hydration technique. Two GBM cell lines and non-cancerous astrocytes were used for the biological evaluation of the tested nanoformulations. The cytotoxicity experiments were performed using the MTT assay, whereas flow cytometry-based analysis assessed the effect of the liposomes on apoptosis, cell cycle distribution, and oxidative stress. To determine the impact of the tested nanoformulations on Nrf2, Wnt/β-catenin, and NF-κB signaling pathways, qPCR, Western blot and ELISA techniques were used. **Results**: The findings of this study demonstrate that liposomal nanoformulations containing CBD, CELE, and DMC exhibit significant anti-GBM activity, particularly through the induction of apoptosis and oxidative stress and modulation of the key signaling pathways. Although no clear synergistic/additive effects were observed between CBD and CELE or DMC when co-loaded in nanoformulations, the combination of CBD and CELE effectively suppressed Wnt/β-catenin and NF-κB signaling and activated the Nrf2 pathway. These results support the therapeutic potential of liposome-based co-delivery of CBD and CELE in GBM therapy. However, further in vivo studies are warranted to determine these nanoformulations’ translational relevance and clinical applicability.

## 1. Introduction

Glioblastoma multiforme (GBM) is an expeditiously progressing brain malignancy, classified by the World Health Organization (WHO) as a grade 4 *IDH*-wild-type glioma based on its molecular and histological pathogenesis [1]. The estimated occurrence of GBM is approximately 3.7 cases per 100,000 individuals, with a 5-year survival of 3–4% [2]. The high mortality associated with GBM stems from its treatment resistance, its substantial infiltration into surrounding tissues, the limited effectiveness of surgical resection, and its pronounced intratumor heterogeneity [3].

In light of the poor clinical outcomes, investigating novel treatment paradigms is justified. Lipid-based particles such as liposomes, have gained attention due to their biodegradability, biocompatibility, ease of preparation, and the ability to incorporate both lipid-soluble and water-soluble agents [4,5]. Clinical data have shown an acceptable safety profile of pegylated liposomal doxorubicin in GBM patients, yet the response patterns remain inconsistent [6]. Development of liposomal delivery requires improvements and deeper exploration to obtain positive outcomes in the suppression of tumor growth. Moreover, nanocarrier-based delivery systems offer the incorporation of unstable anti-cancer compounds such as cannabidiol (CBD), facilitating more effective and predictable drug release [7].

CBD, as a *Cannabis sativa* non-intoxicating bioactive cannabinoid, exerts recognized pro-apoptotic effects and has been shown to induce ferroptosis and autophagy in glioma cells [8,9]. CBD affects multiple molecular targets, including direct or indirect interaction with endocannabinoid system receptors such as cannabinoid receptors (CB1, CB2) or transient receptor potential vanilloid (TRPV2, TRPV4), endoplasmic reticulum (ER) stress induction, and alteration in growth-related pathways [8,10,11]. Also, CBD combined with the standard glioma chemotherapeutic temozolomide (TMZ) demonstrated synergistic cell-killing responses in 1–10 µM (for CBD) and 30 µM (for TMZ) concentrations in GBM T98G cells [12]. The literature reports various combination therapies involving CBD administration with other cannabinoids resulting in the so-called “entourage effect”, as well as with chemotherapeutics or other anti-cancer agents demonstrating synergistic or additive interaction [13,14]. Another interesting anti-GBM agent is celecoxib (CELE), the selective cyclooxygenase-2 (COX-2) inhibitor, and its dimethyl-analog, 2,5-dimethylcelecoxib (DMC) [15]. The pharmacological interactions between CBD and CELE or CBD and DMC when co-administered remain unexplored, yet it may be a promising approach in oncology, also taking into consideration the recently discovered evidence suggesting the anti-depressant and anti-anxiolytic-like effects of CBD-CELE dual therapy [16].

Sustaining proliferative signaling represents a fundamental hallmark of cancer critically implicated in the unregulated expansion of glioma cells [17]. Molecular abnormalities are frequently observed in PI3K/Akt, RAS/MAPK/ERK, and Wnt/β-catenin pathways, providing targets for therapeutic interventions [18]. Specifically, Wnt/β-catenin axis contributes to self-renewal and supports cancer stem cell-driven recurrence [19]. Its downregulation has been linked to increased susceptibility to TMZ, impeding epithelial–mesenchymal transition (EMT), invasiveness, and metastasis [20,21]. Moreover, the dynamic interplay between Wnt/β-catenin and NF-κB signaling affects inflammation-driven carcinogenesis [22,23]. Dysregulated NF-κB activity facilitates immune escape, proliferation, and neovascularization mediated by numerous target genes, proteins, and inflammatory modulators such as tumor necrosis factor α (TNFα), vascular endothelial growth factor (VEGF), COX-2, and a broad array of interleukins (IL), cytokines, and chemokines, thereby driving tumor aggressiveness [24,25]. The third intriguing, druggable avenue lies in Nrf2-driven redox homeostasis and oxidative stress dynamics. Studies highlight the benefits of Nrf2 knockdown in terms of radiotherapy re-sensitization, but evidence also exists about the beneficial role of Nrf2 agonists in cancer treatment [26,27]. Drugs or drug combinations modulating the above-mentioned signaling pathways are therefore promising candidates for novel anti-GBM therapeutics, while novel nanoformulations might additionally improve their effectiveness.

Thus, in this work, we aimed to design and evaluate in the context of anti-GBM effectiveness, five liposomal nanoformulations encapsulating CBD, CELE, and DMC alone or in combination, i.e., CBD with CELE and CBD with DMC; moreover, empty liposomes were also evaluated. Various parameters, including vesicle size, size distribution, zeta potential, and the anti-tumor efficacy against GBM T98G and U-138 MG cells and human astrocytes (HA), were evaluated to estimate the therapeutic efficacy and safety. We report the biological activity of these liposome-based nanocarriers, showing their cytotoxicity and impact on apoptosis, cell cycle distribution, reactive oxygen species generation, and Nrf2, Wnt/β-catenin, and NF-ĸB signaling pathways. According to our best knowledge, our study is the first to evaluate the above-mentioned drug combinations in the context of GBM cells.

## 2. Materials and Methods

### 2.1. Chemical Compounds and Reagents

1-Palmitoyl-2-oleoyl-sn-glycero-3-phosphocholine (POPC) and 1,2-dioleoyl-3-trimethylammonium-propane (DOTAP) were purchased from Avanti Polar Lipids (Birmingham, AL, USA). Cannabidiol (CBD) was purchased from Medcolcanna Organics Inc., Distrito Especial, Colombia; celecoxib (CELE) and 2,5-dimethylcelecoxib (DMC) were obtained from Merck KGaA (Darmstadt, Germany).

### 2.2. Cell Line Culture

Human astrocytes (HA) were cultured in astrocyte medium supplemented with fetal bovine serum (FBS), astrocyte growth supplement (AGS), and penicillin/streptomycin (P/S) solution—all purchased from ScienCell Research Laboratories (Carlsbad, CA, USA). Human GBM U-138 MG and T98G cell lines were obtained from American Type Culture Collection (ATCC) and were cultivated in Eagle’s Minimum Essential Medium (EMEM) (Gibco, ThermoFisher Scientific, Waltham, MA, USA) containing 10% FBS and 1% P/S solution. Cells were incubated in an atmosphere of 5% CO_2_ at 37 °C.

### 2.3. Liposome Preparation

Liposomes were formulated following the method described in the literature [28] using a blend of 1-palmitoyl-2-oleoyl-sn-glycero-3-phosphocholine (POPC) and 1,2-dioleoyl-3-trimethylammonium-propane (DOTAP) in an 8:2 molar ratio. CBD, CELE, DMC, and their combinations were incorporated at molar ratios of 0.1:8:2 (CBD/CELE/DMC:POPC:DOTAP), 0.05:0.05:8:2 (CBD:DMC:POPC:DOTAP), and 0.05:0.05:8:2 (CBD:CELE:POPC:DOTAP). The resulting liposome preparations contained final concentrations of 31.4 µg/mL CBD, 38.1 µg/mL CELE, 39.5 µg/mL DMC, 6 080.6 µg/mL POPC, and 1 397.1 µg/mL DOTAP, with combined formulations containing 15.7 + 19.8 µg/mL CBD + DMC and 15.7 + 19.1 µg/mL CBD + CELE, respectively. In brief, the active compounds and lipids were dissolved in organic solvents at the designated molar proportions. The solvent was removed from the mixture under reduced pressure using two sequential steps: rotary evaporation under high vacuum for 30 min and further drying under vacuum for 2 h. The resulting thin lipid film was then rehydrated in PBS. Small unilamellar vesicles (SUVs) or large unilamellar vesicles (LUVs) were prepared using the Avanti^®^ Polar Lipids Mini Extruder system (Merck KGaA, Darmstadt, Germany) equipped with a polycarbonate membrane of 100 nm pore size.

### 2.4. Liposome Size, Polydispersity Index, and Zeta Potential Measurement

The size of liposomes, polydispersity index (PDI), and zeta potential were determined using a Zetasizer Nano ZS (Malvern Instruments, Malvern, UK), which operates on the principle of dynamic light scattering (DLS) and electrokinetic light scattering (ELS). All measurements were conducted in triplicate. For optimal particle concentration, 10 µL of the liposome suspension was diluted in 10 mL of water. Measurements were carried out in U-shaped cuvettes equipped with gold electrodes.

### 2.5. MTT Viability Assay

Cytotoxicity exerted by the prepared liposomes was evaluated based on the MTT 3-(4,5-dimethylthiazol-2-yl)-2,5-diphenyltetrazolium bromide assay. A total of 10,000 cells/well were seeded in 96-well plates non-coated for U-138 MG and T98G cells and coated with poly-D-lysine for HA. The following day, cells were exposed to liposome treatment at concentration range 1–50 μM for 24 and 48 h of incubation at 37 °C. For the negative control, cells remained in the culture medium. The assay was executed by the addition of 200 μL of MTT diluted solution to PBS-washed cells, followed by 4 h of incubation. Using Infinite M200 microplate reader (TECAN, Grödig, Austria), the absorbance measurement at 570 nm wavelength of formazan dissolved in acidic propanol was used to quantify the metabolic activity of cells, reflecting their viability. The percentage values are presented relative to untreated control cells (100%).

### 2.6. Extraction of Cytosolic and Nuclear Fractions

U-138 MG cells at 200,000 density were seeded on 6-well plates. After 24 h, nanoformulations were added at 1, 5, and 10 μM concentrations, and the cells were incubated for 24 h. Upon harvesting, cells underwent fractioning on ice with the Nuclear/Cytosol Fractionation Kit (Abcam, Cambridge, UK) according to the manufacturer’s instructions. The Lowry method determined total protein concentration in samples based on absorbance measurement.

### 2.7. RNA Extraction and cDNA Synthesis

RNA was isolated from U-138 MG cells previously seeded into 6-well plates (200,000 cells/well) and incubated with liposomal formulations at 1, 5, and 10 μM concentrations for 24 h. The total RNA extraction was performed using the Universal RNA Purification Kit (EURx, Gdańsk, Poland) based on the company’s guidelines. The quantity and quality of RNA were quantified using a NanoDrop^®^ spectrophotometer (ThermoFisher Scientific, Waltham, MA, USA). Next, the extracted RNA was reverse-transcribed to cDNA using RevertAid First Strand cDNA Synthesis Kit (ThermoFisher Scientific, Waltham, MA, USA).

### 2.8. Quantitative Real-Time PCR (qRT–PCR)

To evaluate the expression of the genes of interest, we used the SYBR Green PCR Mastermix (ThermoFisher Scientific, Waltham, MA, USA). Gene-specific primers included *Nrf2* (forward 5′-ATTGCTACTAATCAGGCTCAG-3′; reverse 5′-GTTTGGCTTCTGGACTTGG-3′), *NQO1* (forward 5′-CAATTCAGAGTGGCATTC-3′; reverse 5′-GAAGTTTAGGTCAAAGAGG-3′), *SOD1* (forward 5′-CGACAGAAGGAAAGTAATG-3′; reverse 5′-TGGATAGAGGATTAAAGTGAGG-3′), *CTNNB1* (forward 5′-GGTGACAGGGAAGACATC-3′; reverse 5′-GACAAAGGGCAAGATTTCG-3′), *BIRC5* (forward 5′-GGACCACCGCATCTCTAC-3′; reverse 5′-CCTTGAAGCAGAAGAAACAC-3′), *AXIN2 *(forward 5′-TAGGTTCTGGCTATGTCTTTGC-3′; reverse 5′-GCCTTCACACTGCGATGC-3′), *c-MYC* (forward 5′-TTACAACACCCGAGCAAG-3′; reverse 5′-AATCCAGCGTCTAAGCAG-3′), *NEDD9* (forward 5′-GAACAAGAGGTATATCAGGTG-3′; reverse 5′-TTGAGTGGTATGAGAAGGAG-3′), *CCND1* (forward 5′-CCCTCGGTGTCCTACTTC-3′; reverse 5′-TCCTCGCACTTCTGTTCC-3′), *p50* (forward 5′-ATCATCCACCTTCATTCTCAA-3′; reverse 5′-AATCCTCCACCACATCTTCC-3′), *p65* (forward 5′-CGCCTGTCCTTTCTCATC-3′; reverse 5′-ACCTCAATGTCCTCTTTCTG-3′), and *COX-2* (forward 5′-CGCCTGTCCTTTCTCATC-3′; reverse 5′-CAGCCCGTTGGTGAAAGC-3′). The polymerase chain reaction (PCR) protocol were comprised of the following forty cycles: (1) denaturation (15 s, 95 °C), (2) annealing (30 s, 56 °C), and (3) extension (30 s, 72 °C). The results were then analyzed using the LightCycler96 version 1.1.0.1320 (Roche, Basel, Switzerland). Each reaction was normalized to *TBP* (TATA box binding protein) (forward 5′-GGCACCACTCCACTGTATC-3′; reverse 5′-GGGATTATATTCGGCGTTTCG-3′) and *PBGD* (porphobilinogen deaminase) (forward 5′-TCAGATAGCATACAAGAGACC-3′; reverse 5′-TGGAATGTTACGAGCAGTG-3′) reporter gene expression, and relative mRNA expression was obtained using the calculation of 2^−ΔΔCt^.

### 2.9. Apoptosis Analysis

The GBM T98G and U-138 MG cells were cultured at a density of 200,000 cells/well in 6-well plates and incubated for 24 h. Liposomes suspended in medium were added to cells at 5 and 10 μM and incubated for 24 h. Liposome-treated and control cells were washed with PBS, trypsinized, and centrifuged. Following Annexin V & Dead Cell kit protocol (Merck KGaA, Darmstadt, Germany), the appropriate amount of cells (around 50,000) was resuspended in 100 µL of culture medium and then the cells were incubated with a mixture of Annexin V and 7-aminoactinomycin D (7-AAD) for 20 minutes. Afterwards, flow cytometry was performed to distinguish between live, early-apoptotic, late-apoptotic, and necrotic cells. Analyses were performed on Muse^®^ Cell Analyzer (Merck KGaA, Darmstadt, Germany). The raw data and plots were obtained using the Muse^®^ 1.4 Software (Merck KGaA, Darmstadt, Germany).

### 2.10. Cell Cycle Distribution Analysis

U-138 MG cells were seeded at 200,000 cells/well on 6-well plates, allowed to attach for 24 h, and then exposed to liposomal formulations loaded with single compounds and combinations at concentrations of 5 and 10 μM for 24 h. Detached cells were double-washed with PBS and fixed in 70% cold ethanol, then stored overnight at a freezer at −20 °C. The assays were performed using Muse^®^ Cell Cycle Kit (Merck KGaA, Darmstadt, Germany) containing RNase A and propidium iodide (PI) to obtain appropriate DNA staining with high specificity. After removing residual ethanol, the cells were incubated with reagents for 30 min without exposure to light, and the data were proceeded using Muse^®^ Cell Analyzer and Muse^®^ 1.4 Analytical Software (Merck KGaA, Darmstadt, Germany).

### 2.11. Oxidative Stress Analysis

To evaluate the pro-oxidative effects of tested formulations on U-138 MG cells we seeded the cells at 200,000 cells/well on 6-well plates and allowed them to attach during the next 24 h. Afterwards, the cells were treated with liposomes at concentration of 5 and 10 μM. Cells incubated with doxorubicin (100 nM) were used as positive control, and untreated cells were the negative control. After 24 h, cells were harvested, washed with PBS buffer, and, after centrifuging, resuspended in Assay Buffer. Next, 10 μL of the cell suspension was mixed with 190 μL of Muse^®^ Oxidative Stress working solution prepared ex tempore by dilution of Muse^®^ Oxidative Stress Reagent in Assay Buffer. The Muse^®^ Cell Analyzer (Merck KGaA, Darmstadt, Germany) detected fluorophore—ethidium bromide—bounded to DNA in cells exhibiting superoxide anions. ROS-positive and ROS-negative cells were analyzed using the Muse^®^ 1.4 Software (Merck KGaA, Darmstadt, Germany), and the obtained data were compared to the negative control.

### 2.12. NF-ĸB and Nrf2 Binding to DNA Analysis

Transcription factor activation of NF-ĸB p50, NF-ĸB p65, and Nrf2 was assessed using the TransAM™ NF-ĸB- and Nrf2 ELISA-based DNA-binding kits (Active Motif, Carlsbad, CA, USA). A total of 2 μL nuclear fractions, diluted in complete lysis buffer, were incubated for 2 h in oligonucleotide-coated 96-well plates. Detection of activated transcription factors required primary antibodies targeting epitopes on NF-ĸB p50, NF-ĸB p65, and Nrf2, exposed after DNA binding. To ensure specificity, extensive washing steps were performed, followed by 1 h incubation with primary antibodies to allow immunoconjugate formation. After additional washing steps, an HRP-conjugated anti-IgG secondary antibody was applied for 1 h. A colorimetric reaction was subsequently developed, and the absorbance was measured at 450 nm with a reference wavelength of 655 nm.

### 2.13. Western Blot Analysis

Before loading on a Mini-PROTEAN^®^ TGX Stain-Free™ Protein Gels (BioRad Laboratories, Hercules, CA, USA), cytosol and nuclear protein extracts were denatured by adding denaturation buffer in a 1:5 volume ratio and heating. The amount of 100 μg total protein content per well was loaded into gels and separated electrophoretically at a constant voltage of 200 V for 37 min. After transferring from gel to Immobilon P membrane (Sigma-Aldrich, St. Louis, MO, USA), non-specific regions were blocked with 10% skimmed milk in DPBS buffer for 2 h at room temperature. The proteins of interest, namely Nrf2, NQO1, SOD1, β-catenin, phospho-β-catenin, p50 (NF-κB), p65 (NF-κB), and COX-2, were conjugated with primary antibodies (Santa Cruz, CA, USA) via overnight incubation. Then, membranes were triple-washed with DPBS and incubated with corresponding secondary antibodies conjugated to alkaline phosphatase (AP) or horseradish peroxidase (HRP). The protein-antibody complexes were visualized using the AP Conjugate Substrate Kit NBT/BCIP and the chemiluminescent HRP substrate of the Clarity ECL Kit (BioRad Laboratories, Hercules, CA, USA), respectively, and BioRad ChemiDoc™ imaging system (BioRad Laboratories, Hercules, CA, USA). Stain-free imaging technology (BioRad Laboratories, Hercules, CA, USA) was used to normalize the bands to total protein [29]. The densitometric quantification of bands was analyzed with ImageLab 6.1.0 (BioRad Laboratories, Hercules, CA, USA).

### 2.14. Statistical Analysis

Statistical analysis was performed using GraphPad Prism 9.2.0 (GraphPad Software, San Diego, CA, USA), applying one-way ANOVA with Dunnett’s post hoc test. The statistically significant results were considered for *p* < 0.05 and *p* < 0.01 compared with the untreated group.

## 3. Results

### 3.1. Homogenous Particle Size and Positive Surface Charge of Liposomal Formulation

**Figure 1:**
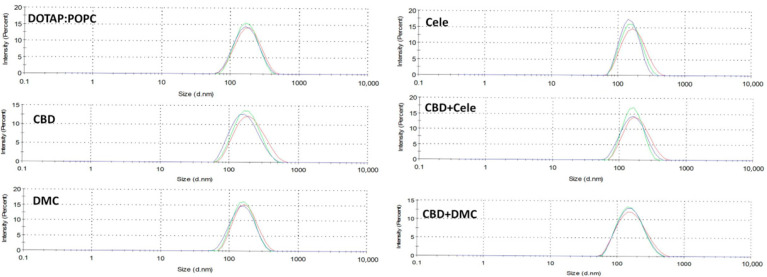
The particle size of liposomal formulations containing CBD, DMC, and CELE and their mixtures (CBD + CELE and CBD + DMC) is characterized using dynamic light scattering (DLS).

The liposomal formulations were characterized in terms of particle size, polydispersity index (PDI), and zeta potential to assess the homogeneity and colloidal stability of the nanoformulations. Measurements were performed in triplicate immediately prior to conducting the in vitro assays to ensure repeatability of measurements. The results are summarized in Figure 1 and Table 1.

The mean particle size of the formulated liposomes was less than 185 nm, exhibiting a narrow size distribution indicative of a homogeneous vesicle population. PDI values determined for the liposomes with drugs were found to be below 0.21. While FDA guidelines [30] emphasize the relevance of achieving a uniform size distribution in liposomal formulations, they do not specify an explicit threshold. Nonetheless, according to the literature reports, liposome suspensions exhibiting PDI values ≤ 0.3 are generally considered to possess a homogeneous size distribution [31,32]. Accordingly, the liposomal formulations obtained in this study can be classified as having a homogeneous vesicle population.

The zeta potential reflects the surface charge characteristics of nanoparticles, which may exhibit cationic, anionic, or neutral properties. A zeta potential exceeding +30 mV or lower than −30 mV is typically regarded as an appropriate threshold for ensuring the colloidal stability of phospholipid vesicles [33,34]. In this study, the obtained liposomes demonstrated a stable cationic surface charge.

### 3.2. Liposomal Celecoxib Exerts High Cytotoxicity Towards GBM Cell Lines

**Figure 2:**
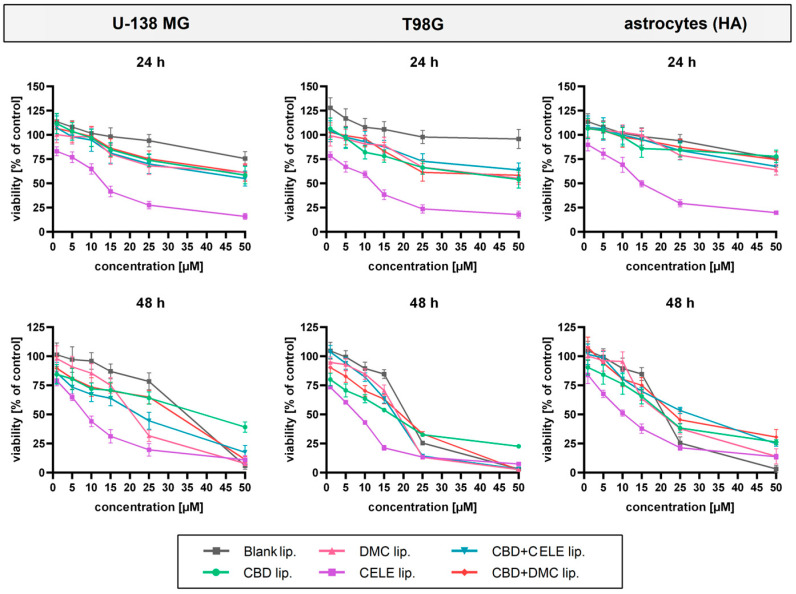
The results of the MTT assay following 24 h and 48 h of exposure of U-138 MG GBM, T98G GBM, and human astrocyte (HA) cell lines to blank liposomes and liposomes loaded with CBD, DMC, CELE, and their combinations. The controls were considered as untreated cells. Data are presented as mean values ± SEM from three independent experiments.

Using MTT assay, we assessed the cytotoxicity of the formulated nanocarriers on GBM U-138 MG and T98G cell lines as well as a non-cancerous human astrocytes (HA) line. Each cell line underwent treatment with single-loaded carriers (with CBD, CELE, and DMC) at the concentration range of 1–50 µM and with combination-loaded carriers corresponding to the same total concentration of compounds, for 24 and 48 h. We considered the untreated viable cells as a control group; additionally, we tested blank liposomes in corresponding amounts of loaded carriers. Figure 2 illustrates the results of the performed assay showing a dose-dependent reduction in cell viability, with the strongest effect observed for the CELE-loaded nanoformulation. The latter one significantly reduced the number of living cells in all tested cell lines in both time points, but particularly following 48 h of incubation. We observed reduction of cell viability exceeding 50% when the liposomes containing 10 µM concentration of CELE were used. 

Slightly lower cytotoxicity was induced in HA in response to both drug-containing as well as blank liposomes; at concentrations below 10 µM the toxicity was minimal, which determined the maximum carrier dose for the subsequent experiments. Data from the MTT assay served to calculate the half maximal inhibitory concentration (IC_50_) of liposomal therapy using linear interpolation method. As presented in Table 2, the encapsulated compounds exerted more potent cytotoxicity toward the T98G line than the U-138 MG line. Although astrocytes exhibited vulnerability to the formulations, the IC_50_ values were higher in the non-cancerous cells than in the malignant T98G cell line after exposure to combination-loaded particles.

### 3.3. Increased Early Stage of Apoptosis in U-138 MG and T98G GBM Cells Induced by CBD-Loaded and CBD + CELE-Loaded Liposomes

**Figure 3:**
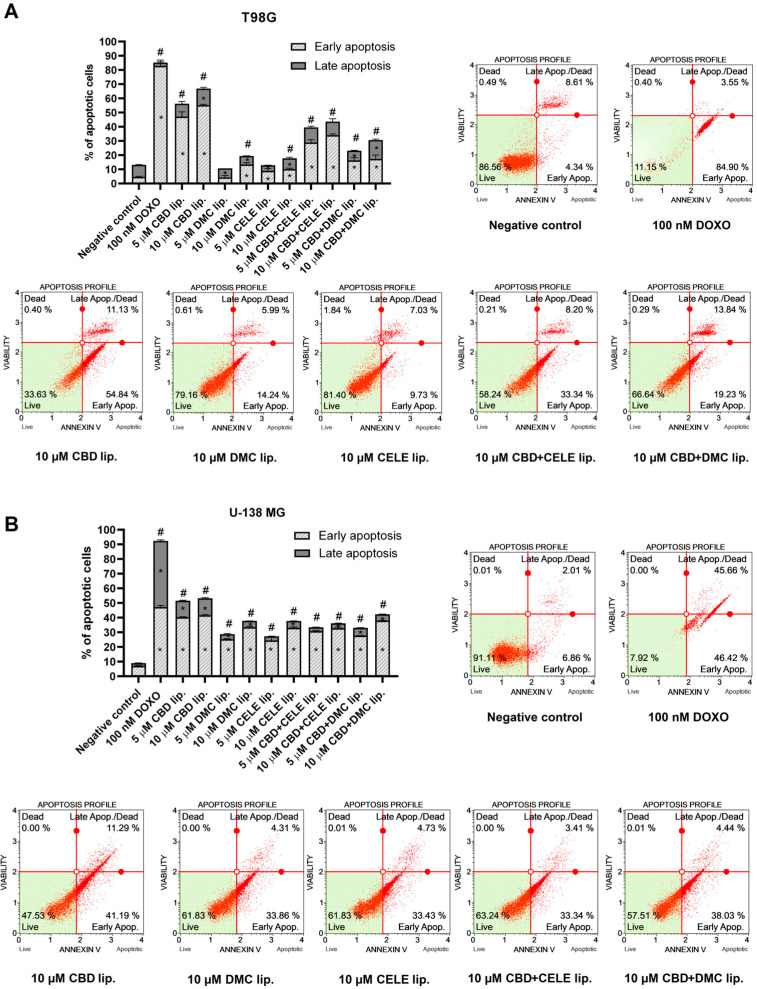
Apoptosis of T98G (**A**) and U-138 MG (**B**) cells treated with liposomal nanoformulation of CBD, DMC, and CELE and their combinations and blank liposomes for 24 h. Doxorubicin (DOXO) served as a positive control, and non-treated cells were the negative control of the assay. Values are expressed as mean ± SEM from two independent experiments. An asterisk (*) indicates the values significantly different from the untreated control cells with *p* < 0.05. A hashtag (#) above the bar indicates statistically significant differences compared to the untreated control for total apoptotic cells. Representative histograms are also presented.

Next, we investigated whether the cytotoxic effect of the nanoformulations was interlinked with apoptosis induction. Thus, following a 24 h treatment, we analyzed phosphatidyl serine (PS) translocation with annexin V and cell membrane disruption with 7-AAD using flow cytometry. In both malignant cell lines, liposomes significantly increased the proportion of early apoptotic cells. In T98G cells, treatment with 10 µM CBD liposomes resulted in nearly 70% total apoptotic cells (Figure 3A). Meanwhile, in U-138 MG (Figure 3B), 5 and 10 µM CBD-loaded liposomes led to 51% and 53% of cells staining positive for annexin V, respectively. Although CELE-loaded liposomes exerted high cytotoxicity in both cell lines, a twofold higher percentage of apoptotic cells was noted in U-138 MG cells than in T98G cells. A significant increase in cells in early apoptosis was observed following treatment with the mixtures of CBD and CELE as well as CBD and DMC encapsulated in the lipid carrier, yet it seems not to be a more effective inducer of programmed cell death than single-loaded CBD. Interestingly, the sensitivity to the combination was cell line-dependent, as U-138 MG responded with stronger apoptosis induction to CBD + CELE and CBD + DMC treatment. Given that U-138 MG cells represent a more aggressive GBM model with higher resistance to chemotherapy and radiotherapy [35], this cell line was chosen for further investigation in the subsequent stages of the study.

### 3.4. Enhanced ROS Production and Activation of Nrf2 Antioxidant Pathway After CBD-Liposomes and CBD + CELE-Liposomes Treatment

**Figure 4:**
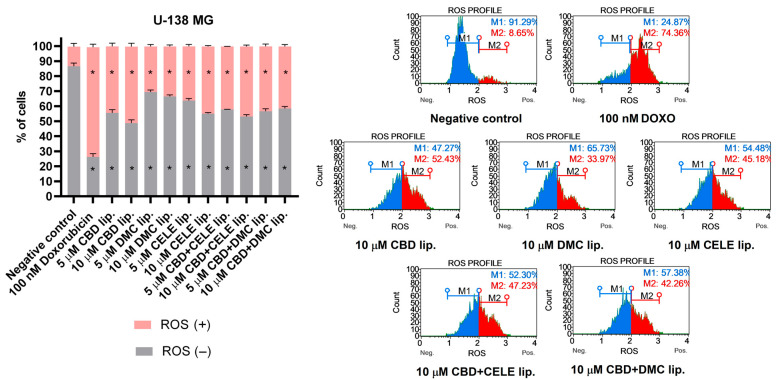
ROS profile was obtained in U-138 MG cells after 24 h treatment with CBD, DMC, CELE, CBD + CELE, and CBD + DMC liposomes. Non-treated cells and DOXO (100 nM) served as negative and positive control of the assay, respectively. ROS (+) and ROS (−) indicate cells with detected and undetected superoxide radicals, respectively. Values are expressed as mean ± SEM from two independent experiments. An asterisk (*) indicates the values significantly different from the untreated control cells with *p* < 0.05. Representative histograms are also presented.

To thoroughly define the impact of the nanoformulations loaded with CBD, CELE, DMC, and their combinations on redox homeostasis, we investigated the cellular redox state by measuring ROS levels as well as the antioxidant response of cells via engagement of the Nrf2 pathway. Compared to untreated cells, all compound-loaded liposomes significantly increased oxidative stress in U-138 MG cells (Figure 4). After 24 h of exposure to 5 µM and 10 µM CBD liposomes, 44.26 ± 2.14% and 50.85 ± 2.23% of the cell population, respectively, exhibited intracellular ROS. Combination treatment led to the accumulation of superoxide anions at levels similar to those induced by 10 µM CELE liposomes administered alone, with approximately 45% of ROS-positive cells.

**Figure 5:**
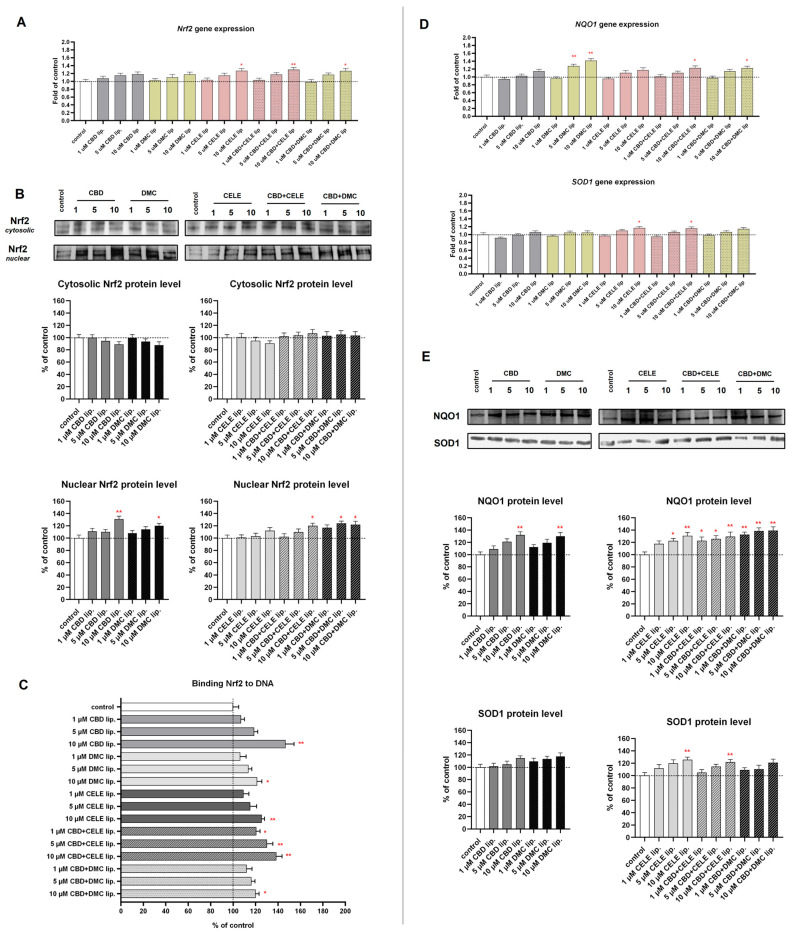
The effect of the liposomes loaded with CBD, DMC, CELE, and their combinations on the *Nrf2* mRNA expression (**A**); translocation from cytosol to nuclei (**B**); the Nrf2–DNA binding capability (**C**); mRNA and protein level of Nrf2 target genes *NQO1* and *SOD1* (**D**,**E**) in U-138 MG cells after 24 h incubation. Data (mean ± SEM) from two separate experiments are presented as fold of control, which are defined as 100% for DNA binding capability and protein expression. Representative immunoblots are shown to analyze the cytosolic and nuclear Nrf2 protein levels (**B**) and cytosolic NQO1 and SOD1 protein (**E**). Bands are presented in the same sequence as the corresponding bars in the graph. A single asterisk (*) and double asterisk (**) denote statistical significance relative to the untreated control cells with *p* < 0.05 and *p* < 0.01, respectively.

As Nrf2 serves as a key oxidative stress sensor, we assessed this pathway’s activation in response to treatment. As shown in Figure 5C, treatment with CBD, DMC, CELE, and their combinations significantly increased Nrf2 DNA-binding activity dose-dependently. CBD liposomes alone induced both ROS accumulation and robust activation of the Nrf2 pathway, with enhanced DNA-binding of transcriptional factor by 46% and significant upregulation of antioxidant protein NQO1 (Figure 5E). The application of both combination-loaded liposomes at 10 µM concentration enhanced Nrf2 transcription factor nuclear translocation (Figure 5B). Notably, the increased nuclear presence of Nrf2 correlated with elevated DNA-binding activity following treatment with CBD + CELE combination liposomes at all tested concentrations, indicating post-translational regulation. Interestingly, an upregulation of the *Nrf2* gene and Nrf2 targets *NQO1* and *SOD1* and their encoded proteins was observed (Figure 5A,D,E).

### 3.5. Treatment with CBD-, CELE-, and Mixture-Loaded Liposomes Halts the GBM U-138 MG Cells in S Phase

**Figure 6:**
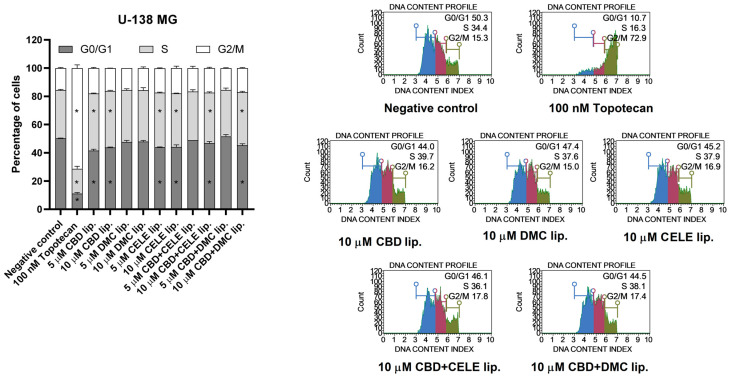
Cell cycle analysis of U-138 MG cell line after 24 h of treatment with CBD, DMC, CELE, CBD + CELE, and CBD + DMC liposomes using the Muse Cell Cycle Kit. Non-treated cells and topotecan (100 nM) served as negative and positive control of the assay, respectively. Values are expressed as mean ± SEM from two independent experiments. An asterisk (*) indicates the values significantly different from the untreated control cells with *p* < 0.05. Representative histograms are also presented.

To investigate the link between liposome-mediated signaling pathway modulation and cellular functions, their effect on cell cycle distribution was analyzed (Figure 6). The flow-cytometry measurement of DNA content revealed that CBD-loaded liposomes, CELE-loaded liposomes, and the liposomes loaded with both the above-mentioned compounds as well as CBD + DMC-loaded liposomes induce changes in cell cycle distribution; after 24 h treatment we observed mild though statistically significant decrease in the number of cells in the G0/G1 phases, accompanied by an increase in the number of cells in the S phase. Consistent with expectations, topotecan, an anti-cancer drug employed as a reference compound, led to an accumulation of cells in the G2/M phase, a response that was not observed following treatment with liposomes.

### 3.6. Wnt/β-Catenin Pathway Inhibition in Response to CBD + CELE Entrapped in Liposomes

**Figure 7:**
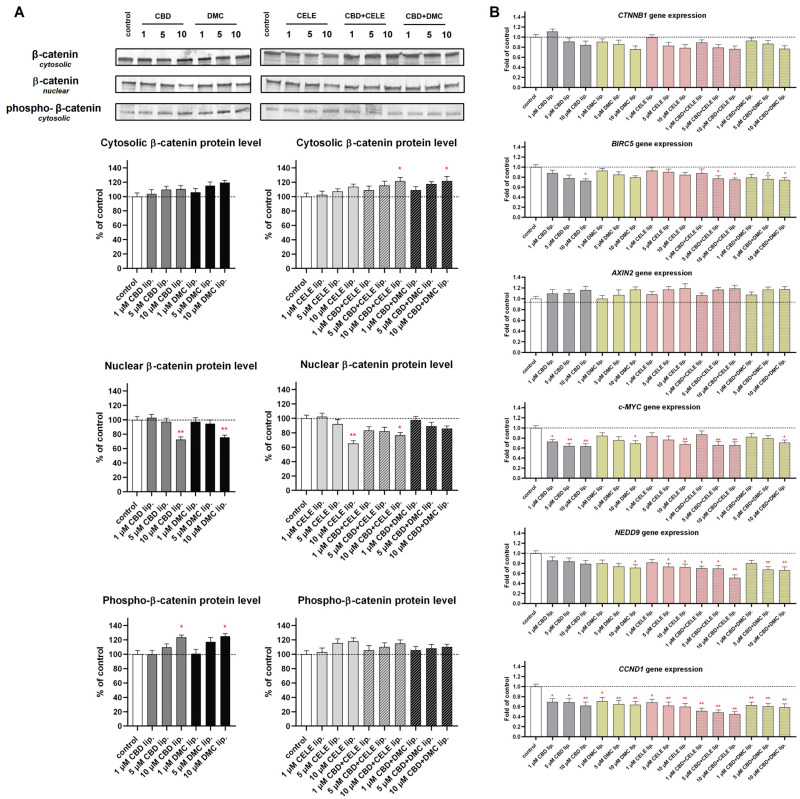
The effect of the liposomes loaded with CBD, DMC, CELE, and their combinations on the *β*-catenin translocation from cytosol to nuclei and phospo-β-catenin level in cytosol (**A**) and Wnt/*β*-catenin target genes *CTNNB1*, *BIRC5*, *AXIN2*, *c-MYC, NEDD9*, and *CCND1* mRNA expression (**B**) in U-138 MG cells after 24 h incubation. Data (mean ± SEM) from two separate experiments are presented as a fold of control, which are defined as 100% for protein expression. Representative immunoblots are shown for the analysis of the cytosolic and nuclear of the *β*-catenin protein levels and the cytosolic phospho-*β*-catenin protein levels (**A**). Bands are presented in the same sequence as the corresponding bars in the graph. A single asterisk (*) and double asterisk (**) denote statistical significance relative to the untreated control cells with *p* < 0.05 and *p* < 0.01, respectively.

Next, we analyzed the impact of the studied nanoformulations on the Wnt/β-catenin pathway (Figure 7A,B). The results of our study revealed that the cytosolic level of β-catenin increases after treatment with both combination-loaded liposomes at the 10 µM concentration. In the case of an entrapped combination of CBD + CELE (10 µM), we also observed a decrease in the nuclear level of β-catenin, suggesting Wnt pathway inhibition.

A similar result regarding the decrease in β-catenin in the nucleus was found as a result of 10 µM CBD, 10 µM DMC, and 10 µM CELE but all used independently. The upregulated level of phospho-β-catenin was observed after treatment with 10 µM CBD and 10 µM DMC, suggesting Wnt pathway inhibition. Together with the protein translocation and phosphorylation of β-catenin, we also analyzed the mRNA level of this key Wnt pathway mediator (encoded by the *CTNNB1* gene) as well as the mRNA levels of the major Wnt pathway target genes *BIRC5*, *AXIN2*, *c-MYC*, *NEDD9*, and *CCND1*. In general, we observed downregulation of the *BIRC5* gene after the treatment with 10 µM liposomal CBD, and both liposomal CBD + CELE, and CBD + DMC (both in 5 µM and 10 µM concentration). The level of *AXIN2* mRNA remained generally unchanged. As far as *c-MYC* is concerned, CBD in all analyzed concentrations downregulated its transcript level; so also 10 µM DMC and 10 µM CELE. The nanoformulations containing CBD + CELE (in both 5 and 10 µM concentration) and 10 µM CBD + DMC exerted similar action. Regarding the expression of *NEDD9*, the downregulatory effect was observed after treatment with 10 µM DMC but also CELE in the whole range of tested concentrations, including independent treatment and co-treatment with CBD. A similar effect was also noticed in the case of CBD + DMC in both tested concentrations. As far as *CCND1* is concerned, significantly lower mRNA levels were observed after the treatment with all analyzed nanoformulations.

### 3.7. Reduced NF-κB Pathway Activation Particularly upon CBD + CELE-Loaded Liposomes Treatment

**Figure 8:**
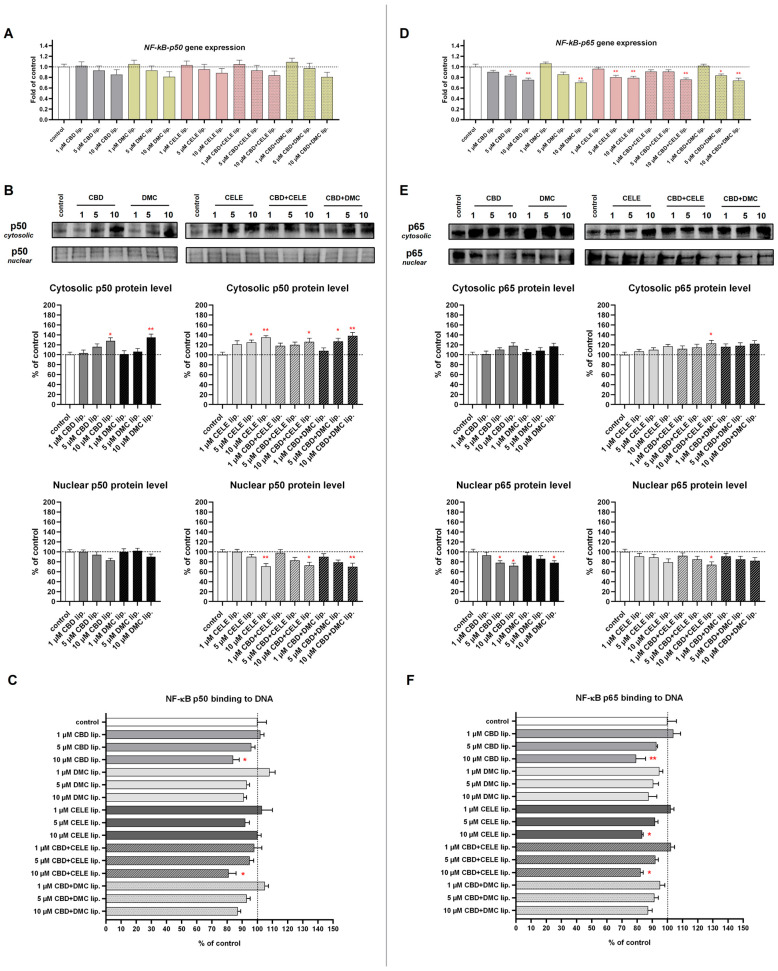
The effect of the liposomes loaded with CBD, DMC, CELE, and their combinations on the *p50* and *p65* mRNA expression (**A**,**D**); translocation from cytosol to nuclei of p50 and p65 (**B**,**E**); and p50 and p65–DNA binding capability (**C**,**F**) in U-138 MG cells after 24 h incubation. Data (mean ± SEM) from two separate experiments are presented as fold of control, which are defined as 100% for DNA binding capability and protein expression. Representative immunoblots are shown to analyze the cytosolic and nuclear of NF-κB subunits p50 and p65 protein levels (**B**,**E**). Bands are presented in the same sequence as the corresponding bars in the graph. A single asterisk (*) and double asterisk (**) denote statistical significance relative to the untreated control cells with *p* < 0.05 and *p* < 0.01, respectively.

The inflammatory tumor microenvironment drives resistance to applied therapies; thus, reducing pro-inflammatory mediators in cancer cells may improve treatment outcomes [36]. The NF-κB p50 DNA-binding level significantly decreased in response to 10 µM CBD and 10 µM CBD + CELE liposomes (Figure 8C). Also, Western blot of NF-κB subunit p50 indicated significant decrease in the nuclear levels by over 20% after treatment with CELE- and mixture-loaded liposomes (Figure 8B). Consistent with these observations, the treatment with encapsulated CBD, CELE, and their combinations led to significant suppression of NF-κB p65 DNA-binding activity, as determined by ELISA (Figure 8F). Western blot analyses further confirmed reduced nuclear localization of the p65 subunit under liposomal CBD, DMC, and combined CBD + CELE treatment (Figure 8E). Moreover, *NF-κB-p65* (*RelA*) gene expression was significantly downregulated following exposure to the formulations, aligning with the diminished transcriptional activity (Figure 8D). These results support a dual mechanism of NF-κB inhibition at both transcriptional and post-translational levels.

**Figure 9:**
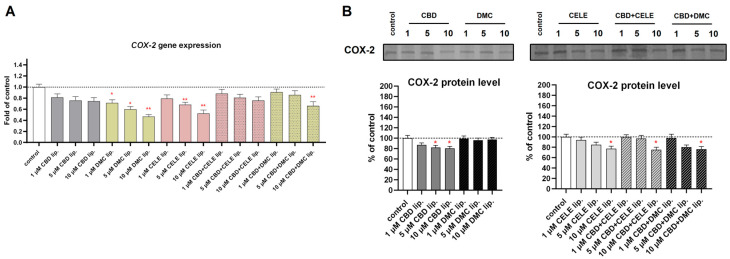
The effect of the liposomes loaded with CBD, DMC, CELE, and their combinations on the *COX-2* mRNA expression (**A**) and the cytosolic COX-2 protein level (**B**) in U-138 MG cells after 24 h incubation. Data (mean ± SEM) from two separate experiments are presented as fold of control, which are defined as 100% for protein expression. Representative immunoblots for the cytosolic COX-2 protein levels are shown. Bands are presented in the same sequence as the corresponding bars in the graph. A single asterisk (*) and double asterisk (**) denote statistical significance relative to the untreated control cells with *p* < 0.05 and *p* < 0.01, respectively.

The COX-2-positive U-138 MG line is characterized by overexpression of this prostaglandin-synthetizing enzyme that plays a role in cancer’s aggressive phenotype. As we expected, after CELE-loaded liposome (10 µM) treatment, the cytosolic levels of COX-2 decreased by 20%, and *COX-2* mRNA expression was reduced by 50% (Figure 9A,B). The drop in COX-2 protein expression was observed following CBD treatment in a dose-dependent manner. Also, CBD + CELE and CBD + DMC downregulated COX-2 protein expression, while the mRNA level dropped only in response to 10 µM CBD + DMC liposomes.

## 4. Discussion

In this study, liposomal nanoformulations containing CBD, DMC, and CELE were obtained, and their anti-tumor effects were demonstrated. The rationale for encapsulating CBD, DMC, and CELE within nanoparticles was to enhance their solubility and stability. We obtained cationic liposomal formulations with a narrow size distribution and a diameter below 180.1 nm. The pharmacokinetic behavior of liposomes, including systemic circulation, clearance by the mononuclear phagocyte system (MPS), extravasation into the tissue interstitium, interstitial transport through the extracellular matrix, as well as cellular uptake and intracellular trafficking, is strongly influenced by particle size [31,37]. Since our nanoparticles were smaller than 200 nm, we can assume they might exhibit reduced opsonization by serum proteins and diminished clearance by the MPS [38]. Additionally, our choice for cationic liposomes was justified by the fact that cationic vesicles are capable of engaging in electrostatic interactions with the negatively charged glycocalyx of the luminal surface of the BBB, thereby facilitating adsorptive-mediated transcytosis. This mechanism has been extensively explored in numerous studies aiming to enhance the delivery of therapeutics for brain tumor treatment [39]. Moreover, the data in this study support the use of DOTAP:POPC liposomes as an alternative to DMSO solutions in in vitro studies. Eliminating the need for DMSO as a potentially toxic solvent reduces the likelihood of solvent-induced effects and allows for obtaining a more accurate understanding of the substances being investigated [40].

Our choice for the analysis of CBD, CELE, and DMC, all alone and in combination, was justified by the fact that a growing amount of in vitro and in vivo studies indicate exceptionally good anti-cancer properties of these substances, among others tested, providing rationale for in vivo studies, including human clinical trials.

The most spectacular results so far were obtained for CBD in the context of GBM patients’ survival, as presented by the pivotal phase Ib clinical trial (Part1—NCT01812603, Part 2—NCT01812616). The study aimed to assess the safety, tolerability, and efficacy of a sublingual spray, Sativex^®^ (containing 27 mg/mL THC and 25 mg/mL CBD), in combination with dose-intense temozolomide (DIT) therapy in patients with recurrent GBM [41]. Overall, the trial demonstrated that the personalized dosing regimen of Sativex^®^ in combination with DIT has good safety and tolerability in patients with recurrent GBM, potentially contributing to an increase in survival rates (the first part achieved a 6-month progression-free survival (PFS6) of 16.7% and a 1-year survival rate of 50.0%; the second part had a PFS6 of 33.3% and a 1-year survival rate of 83.3%). Currently, several clinical trials concerning the treatment of gliomas with CBD (NCT03529448, NCT03246113, NCT05629702, and NCT03607643) are underway.

As far as CELE is concerned, a phase II clinical trial (NCT00504660) evaluating the combination of 6-thioguanine, capecitabine, and CELE with TMZ or lomustine in the treatment of recurrent or progressive anaplastic glioma or GBM in patients who have failed previous treatments showed that these drug combinations do not appear to be more effective than other alkylating agent schedules for patients with recurrent GBM [42]. Additionally, a phase I trial established the safety of combining dose-dense TMZ with CELE [43]. However, a factorial randomized phase II trial in participants with newly diagnosed GBM (NCT00112502) did not demonstrate improved PFS for such a regimen [44]. Thus, there is a need for some improvements in the context of CELE’s use in GBM therapy. Thus, our study hypothesized that encapsulating CELE in liposomal nanoformulation and adding CBD as a booster might increase its efficacy.

DMC is a celecoxib analog that lacks COX-2 inhibitory activity but still exhibits cytotoxic properties. Importantly, studies show stronger anti-tumor properties of DMC as compared to CELE [45]. The anti-glioma potential of DMC has been demonstrated in numerous studies, including ours [46] and that of Gao et al., who demonstrated that DMC inhibits proliferation and the cell cycle and induces apoptosis in GBM by suppressing CIP2A/PP2A/Akt signaling axis [47]. Nevertheless, DMC has not yet reached the level of GBM clinical trials.

As presented above, recent studies have provided evidence that all three substances express very promising anti-glioma properties; however, they also have some limitations. Thus, given that CBD exerts anti-proliferative, cytotoxic, and anti-neoplastic activity, we hypothesized that it may potentiate the therapeutic effects of CELE or DMC and improve the therapeutic effects of both drugs when used as dual therapy. Such a strategy of combinatory treatment is generally appreciated in cancer treatment, as single agents often do not provide satisfactory results [48].

Our study is the first to evaluate the above-mentioned drug combinations in the context of GBM. Interestingly, recently, the effects of CELE, CBD, and their combination were examined in murine models of anti-depressant- and anxiolytic-like behavioral responsiveness, showing that CELE plus CBD produce efficacious antidepressant- and anxiolytic-like effects [16]. These effects, if similar in humans, would be of great value in the context of GBM patients, therefore requiring further investigation. Nevertheless, concerning GBM patients, the anti-cancer potential of these drug combinations is superior as compared to anti-depressant- and anxiolytic properties and therefore was the aim of this study.

Thus, this study investigated the cytotoxic potential of liposomal CBD, CELE, and DMC in three human-derived cell lines, i.e., U-138 MG, T98G (both being TMZ-resistant GBM cell lines), and non-cancerous human astrocytes (HA). The analysis was performed at two time points and showed that all tested nanoformulations influence cell viability of both tumor and normal cells in a concentration- and time-dependent manner. It is important to indicate that GBM cells were found to be more susceptible to the cytotoxic effects of the analyzed nanoformulations as compared to the HA cells, and the concentrations already active towards cancer cells (5 and 10 µM) were safe for the HA cells. This finding paves the way for future in vivo studies of these nanoformulations, in which the safety of non-cancerous brain structures is of primary importance.

Our additional finding was that although we initially hypothesized that CBD might potentiate the effects of CELE, our MTT data did not confirm this assumption. On the contrary, CBD seemed to attenuate the cytotoxicity of CELE when co-loaded into liposomes. On the other hand, our apoptosis evaluation indicated a higher pro-apoptotic potential of the CBD-CELE combination compared to CELE alone (while CBD alone was the most effective inducer of apoptosis). It should also be noted that MTT and apoptosis analyses evaluate two distinct aspects of cellular response to the given treatment—MTT is based on the enzymatic activity of living cells, while apoptosis analysis relies on detecting the presence of phosphatidylserine on the plasma membrane of apoptotic cells, both showing distinct aspects of the anti-cancer effects of an analyzed substance. Additionally, it is important to mention that annexin V staining primarily detects early apoptotic events and does not necessarily indicate irreversible cell death. Therefore, interpretation of apoptosis based solely on annexin V/7-AAD staining may be limited, and in this regard, the MTT assay provides a more reliable measure of overall cell viability.

Nevertheless, in order to clarify the pro-apoptotic effect of the tested nanoformulations, we evaluated the involvement of ROS generation as a response to the treatment. Oxidative stress plays a crucial role in the effectiveness of all anti-cancer treatments, and the ability of a compound to generate massive amounts of ROS can be of great value in therapeutic efficacy. In our study, the highest intracellular ROS levels were observed after the treatment with liposomes containing CBD. Overall, CBD modulates redox function at multiple levels, and a variety of downstream effects are presented in the literature. These pro- and antioxidant functions of CBD may be cell- and model-dependent and may also be influenced by the dose of CBD delivered, the duration of CBD treatment, and the underlying pathology [49]. Regarding ROS production, CBD has been shown to disrupt mitochondrial integrity and induce ROS production and apoptosis in human CD14+ monocytes, breast cancer cells, and GBM cells [49]. In a study by Kim et al., CBD induced ERK activation and ROS production to promote autophagy and ferroptosis in GBM cells [8]. The authors found that CBD significantly elevated the endoplasmic reticulum stress and iron load as well as decreased GSH levels.

In our study, CBD combinations and CELE used as a single agent as well as DMC elevated the ROS production in GBM cells. Our findings are in agreement with other studies, which show that CELE exerts powerful pro-oxidative anti-cancer effects by directly targeting mitochondria to increase ROS production, triggering cancer cell death [50]. In respect to DMC, there is also mounting evidence that one of its major anti-cancer mechanisms is ROS production. Recently, DMC-induced autophagy involving the ROS/JNK axis has been described [51].

The cellular response to oxidative stress is mediated via the Nrf2 pathway. In our study, the analyzed nanoformulations activated the Nrf2 pathway-dependent antioxidant response. We observed upregulation of *Nrf2* mRNA level, nuclear translocation of Nrf2, and increased binding of Nrf2 to its consensus DNA sequence in response to a combination of CBD and CELE co-loaded in liposomes and when CBD was used as a single agent. Our results suggest that CBD + CELE and CBD alone, when delivered in nanoformulations, effectively trigger the antioxidant response. It remains unknown whether or not it is better to activate or inhibit the Nrf2 pathway during GBM treatment. While some studies suggest improved GBM sensitivity to chemotherapy resulting from Nrf2 inhibition, others reveal the potential clinical application of Nrf2 activators in GBM treatment [27,52]. Therefore, the relevance of Nrf2 modulation in the context of GBM remains to be elucidated.

The Wnt/β-catenin pathway is a signaling cascade involved in cell growth, migration, and other cellular processes, and its dysregulation is linked to GBM development. In general, enhanced Wnt/β-catenin pathway activity is a hallmark of stemness and drug resistance, and Wnt/β-catenin activation has been generally associated with decreased survival in GBM patients [53]. Our previous study showed that CELE and DMC counteract the hyperactivated Wnt/β-catenin pathway and COX-2/PGE2/EP4 signaling in GBM cells [46]. In this study, we provide additional evidence that nanoformulations containing CELE and DMC in combination with CBD suppressed the expression of Wnt/β-catenin target genes such as *NEDD9*, *BIRC5*, *c-MYC*, and *CCND1*. The downregulation of the Wnt target genes was accompanied by decreased nuclear translocation of β-catenin (in case of treatment with CBD, DMC, and CELE used independently or CBD + CELE at 10 µM concentration) and increased β-catenin phosphorylation, indicating pathway inhibition (after treatment with CBD and DMC, both when used independently).

The literature data show that cannabinoids are Wnt modulators, but their exact impact on the Wnt pathway depends on the context, especially the cell type (malignant vs. non-malignant) [54]. Specifically, CBD can modulate the activity of GSK3β, a negative regulator of the Wnt/β-catenin pathway, leading to increased Wnt signaling. This interaction may contribute to CBD’s neuroprotective and anti-inflammatory effects in conditions like glaucoma and Alzheimer’s disease [55,56]. On the other hand, in colorectal cancer cells, CBD can reverse EMT by inhibiting the Wnt/β-catenin pathway. CBD suppresses the activation of the Wnt/β-catenin signaling pathway, inhibits the expression of β-catenin target genes such as *APC* and *CK1*, and increases the expression of *Axin1* [57]. Since the Wnt pathway plays a major role in initiating colorectal tumors, most studies regarding Wnt pathway modulators are performed in this cancer model [58]. The data about the role of CBD as a Wnt pathway modulator in GBM are scarce. The study of Esfandiary et al. showed that *Cannabis sativa* extract may repress the amount of β-catenin by increasing the level of AXIN1 [59]. In a study of Nalli et al., six cannabinoids were evaluated for their cytotoxicity on a panel of the cancer cell lines HCT-116, OVCAR, A549, MCF7, PC-3, HepG2, and SH-SY5Y spanning different tumor types (colon, ovary, lungs, breast, prostate, liver, and neuroblastoma) [60]. They found that cannabinoids exhibited potent inhibitory activity against Wnt/β-catenin pathway in liver cancer HepG2 cells. By monitoring the TCF-dependent β–catenin Top-Flash reporter activity, they found that CBD, its 2, 3-epoxy derivative, and cannabivarin decreased β-catenin transcriptional activity in a dose-dependent manner [60].

Our next goal was to analyze the impact of the studied nanoformulations on the NF-κB pathway, a crucial signaling pathway frequently activated in GBM cells and implicated in various aspects of tumor development, including the maintenance of glioma stem cells, invasion, and resistance to treatment. Our study revealed that NF-κB activation was attenuated upon exposure to 10 µM CBD + CELE liposomes. NF-κB-inhibitory properties were also shown by 10 µM CBD, which affected the DNA-binding of NF-κB p50 and p65 subunits. Our findings are in concordance with other studies, which show that CBD and CELE attenuate the NF-κB pathway in GBM cells. In this context, Ahsan et al. explored the inhibitory effect of CELE on decreasing the expression of NF-κB p65 (RelA) and TNFα in the GBM cell line compared to TMZ [61]. They showed that CELE reduces the expression of NF-κB, which is linked to suppression of COX-2, hence reducing the proliferation of GBM cells [61]. In another study, the treatment of GBM U373 and T98G cells with CELE significantly downregulated NF-κB nuclear translocation, NF-κB DNA binding activity, and NF-κB-dependent reporter gene expression. Additionally, CELE suppressed IκBα degradation and phosphorylation and reduced IKK activity in a dose-dependent manner [62]. As far as CBD is concerned, the results of a pharmacogenomics study by Volmar et al. provided interesting evidence showing that CBD converts NF-κB into a tumor suppressor in GBM with defined antioxidative properties. In this context, sensitivity to CBD was observed in a cohort of human primary GBM stem-like cells defined by low levels of ROS, while high ROS content in other tumors blocked CBD-induced GBM stem-like cells death [63].

Overall, our study presents promising anti-GBM nanoformulations loaded with CBD, CELE, DMC, or their combinations. Nevertheless, it must be acknowledged that our findings, even though encouraging, are based solely on in vitro experiments and require in vivo evaluation. Therefore, animal studies are necessary to confirm the ability of the tested liposomal formulations to cross the BBB, specifically targeting GBM cells within the tumor microenvironment and exerting their therapeutic efficacy under physiological conditions. Also, in vivo experiments will offer more relevant insights into the synergistic potential of the compounds delivered via nanocarriers, from a biomedical perspective. This is because the concentration ratios used in the in vitro models may not reflect the actual local concentrations in the tumor microenvironment due to liposome accumulation, local fluid turnover, differential clearance rates, and compound degradation [64]. Thus, in vivo studies are planned as the next stage of our research to validate the translational relevance of the liposomal formulations investigated here.

## 5. Conclusions

The findings of this study demonstrate that liposomal formulations containing CBD, CELE, and DMC exhibit significant anti-GBM activity, particularly through the induction of apoptosis and oxidative stress and modulation of key signaling pathways. Although no clear synergistic/additive effects were observed between CBD and CELE or DMC when co-loaded, the combination of CBD and CELE effectively suppressed Wnt/β-catenin and NF-κB signaling and activated the Nrf2 pathway. These results support the therapeutic potential of liposome-based delivery of CBD and CELE in GBM. However, further in vivo studies are warranted to determine these nanoformulations’ translational relevance and clinical applicability.

## Figures and Tables

**Table 1:** The particle size, polydispersity index (PDI), and zeta potential of the liposomes.

	Particle Size (±SD) [nm]	PDI (±SD)	Zeta Potential (±SD) [mV]
CBD lip.	184.3 ± 22.1	0.16 ± 0.003	38.4 ± 1.91
DMC lip.	180.9 ± 14.3	0.14 ± 0.04	38.4 ± 2.17
CELE lip.	180.8 ± 19.4	0.18 ± 0.11	41.7 ± 0.40
CBD + CELE lip.	176.4 ± 0.2	0.14 ± 0.05	39.3 ± 1.04
CBD + DMC lip.	179.9 ± 9.8	0.21 ± 0.07	41.3 ± 1.79
Blank lip.	182.3 ± 3.3	0.12 ± 0.01	32.0 ± 7.35

**Table 2:** Half maximal inhibitory concentration (IC_50_) for cell lines U-138 MG, T98G, and human astrocytes (HA) treated with liposomes loaded with CBD, DMC, and CELE and their combinations for 24 and 48 h.

	IC_50_ ± SEM [µM]					
	24 h			48 h		
	U-138 MG	T98G	HA	U-138 MG	T98G	HA
CBD lip.	>50	>50	>50	39.1 ± 2.9	16.8 ± 0.7	20.7 ± 1.6
DMC lip.	>50	>50	>50	20.8 ± 0.8	18.6 ± 0.6	20.3 ± 1.3
CELE lip.	13.2 ± 0.7	12.2 ± 0.7	14.9 ± 0.5	8.6 ± 0.6	8.0 ± 0.4	10.4 ± 1.0
CBD + CELE lip.	>50	>50	>50	22.1 ± 2.2	17.8 ± 0.6	28.0 ± 1.6
CBD + DMC lip.	>50	>50	>50	32.0 ± 1.3	19.4 ± 1.0	23.4 ± 1.4
Blank lip.	>50	>50	>50	34.8 ± 1.1	20.9 ± 0.4	20.9 ± 0.6

## Data Availability

Data will be made available if requested.
